# Which Seat Facilitates the Detection of Off-Seat Behaviours? An Inattentional Blindness Test on Location Effect in the Classroom

**DOI:** 10.3389/fpsyg.2022.899696

**Published:** 2022-06-30

**Authors:** Shuqin Cao, Xiuying Wei, Jiangbo Hu, Hui Zhang

**Affiliations:** ^1^School of Special Education, Zhejiang Normal University, Hangzhou, China; ^2^Zi Jinghua School, Hangzhou, China; ^3^Hangzhou Preschool Teachers College, Zhejiang Normal University, Hangzhou, China

**Keywords:** inattentional blindness, location effect, special education, classroom management, off-seat behaviour

## Abstract

Off-seat behaviour refers to students leaving their seats and walking out of a classroom without the teacher noticing. This behaviour occurs in special education for students with certain special needs, which would lead to serious safety problems. This study carried out an inattentional blindness test to explore whether the location of seats in classrooms would impact teachers’ detection rate regarding off-seat behaviours. The participants were 126 pre-service teachers (M_age_ = 18.72 ± 0.723; 92% female) who were invited to perform the primary task of counting students raising their hands up whilst the disappearance of one of the students was introduced as an unexpected occurrence. The results show that peripheral seats were more detectable than the central ones for the teachers to notice the “missing student.” Meanwhile, the left and below oriented seats were more likely to be ignored compared to those that were right and upper oriented. These results suggest the existence of a location effect in the classroom that is associated with teachers’ attention regarding off-seat behaviour. This study has implications for classroom management in terms of arranging students’ seats appropriately to assist in increasing teachers’ identification of this hazard.

## Introduction

Off-seat behaviour has been reported frequently in the education of students with certain special needs, such as autism spectrum disorder (Patterson and Steven, 2009; Yang et al., 2012; Aspiranti et al., 2018). Off-seat behaviour may result in dangerous consequences if teachers fail to detect the “missing student” in a timely manner. Given the dynamic activities and multi-tasks in a classroom that occupy most of a teacher’s attention resource, it is possible for teachers to unconsciously ignore off-seat behaviour in many situations.

Research suggests a low detection rate of unexpected stimuli for participants being occupied by other tasks. Location effect, that is, detection rate varying according to the location of stimuli’s appearance (e.g., centre or margin), is identified in experimental environments (Newby and Rock, 1998; Most et al., 2000). Based on this notion, it is reasonable to propose that off-seat behaviour that occurred in different locations in a classroom may attract teachers’ attention at different levels. This study is designed to use an inattentional blindness (IB) test to explore the location effect in a classroom for teachers’ detection of off-seat behaviours. The purpose of the IB test is to assess an individual’s ability to recognize unexpected events while concentrating on cognitively demanding tasks that need close attention (Mack and Rock, 1998; Simons and Chabris, 1999). The results of the study would increase our understanding of the location effect in classrooms that is related to teachers’ attention. It has implications for classroom arrangement in terms of positioning children appropriately for preventing off-seat behaviours from being ignored.

### Literature Review

The IB test is widely applied in fields of medicine (Dixon et al., 2013; Drew et al., 2013; Hughes-Hallett et al., 2015; Ho et al., 2017), transportation (Pammer and Blink, 2013; Yuan et al., 2021), sports (Furley et al., 2010; Klatt and Nerb, 2021), and safety practices (Castel et al., 2012; Wiseman and Watt, 2015), where people’s attention needs to be divided for addressing multiple tasks. Researchers attempt to investigate how people detect unanticipated occurrences while concentrating on their missions. A consensus in the literature is that individuals’ IB presented at a variety of levels under different conditions. For example, compared to a single task requiring only visual or auditory attention, the extent of IB could be increased when people are exposed to dual tasks requiring both visual and auditory attention (Alsius et al., 2005). Considering the classroom duties that involve extensive visual and auditory devotion, teachers’ IB rate may be maintained at a certain level during teaching.

To date, the application of the IB test in school education is limited, though teachers’ attentional division is crucial for classroom management (Wolff et al., 2016). Research relating to classroom management has identified a series of impact factors, such as the class size (Finn, 2019; Huang et al., 2022), the cognitive load of the teaching task (Prieto et al., 2018; Feldon et al., 2019; Huang et al., 2021), and children’s behaviours (Phillips and Downer, 2017), which are associated with the quality of classroom management, requiring teachers’ attentional division of different degrees. For special education, teachers may encounter a more complicated situation in the classroom (Allam and Martin, 2021). A common cognitively demanding task can be very difficult for students with special needs, and their challenging behaviours are shown more frequently than other students (Lever, 2014; Polirstok, 2015). However, a particularly relevant study on Chinese teachers’ classroom management in special schools found that the teachers’ attention could be more attracted by students’ attention-seeking behaviours (e.g., raising hand up for questions) rather than challenging behaviours [e.g., being violent to peers; Wang et al. (2013)]. All these studies indicate the uncertainty of teachers’ attentional allocation to individual students in a dynamic classroom environment. Given the unobstructive nature of off-seat behaviour, there is a high possibility for teachers to ignore the quiet “missing student.”

The factors that affect people’s attentional division for detecting unexpected stimuli is the main topic in IB tests. The physical features, such as the size and the colour of unexpected stimuli, are found to be associated with people’s detection rates (Mack and Rock, 1998; Koivisto et al., 2004; Qin and Chen, 2011). In Koivisto et al. (2004) study, the participants were assigned a task to name digits that appeared sequentially on a screen while unexpected stimuli including black, red, blue, and green circles appeared shortly at the center of the screen. The results showed that the participants performed worse in perceiving the black circle (8%) than the red/blue circle (61%) and green circle (53%). This study is partially supported by Kelly et al. (2018) who explored stimuli in different colours in an IB test. The researchers found that the detection rates reduced when the luminance of the stimuli’s colour decreased. The size of stimuli is also identified to be associated with detection rates. The larger size of the stimulus, the higher the possibility it is to be detected by people (Mack and Rock, 1998; Chen, 2013). These findings conveyed the message that more conspicuous stimuli tend to catch more of people’s attention, which is in line with our common sense. Nevertheless, the effect of the stimuli’s feature can be significantly impacted by the participants’ expectation. For participants who were given a vague (covert cue) or clear clue (overt cue) to expect a stimulus, the detection rate will be substantially increased regardless of the feature of the stimuli (Mack and Rock, 1998; Ward and Scholl, 2015; Zhang et al., 2022).

A strand of IB research focuses on the location effect of IB, and inconsistent findings have been reported in the field. Some scholars found that centrally positioned stimuli can attract more attention than those in the margin areas because a central position is closer to the fixation point (Newby and Rock, 1998). This claim is endorsed by the studies that demonstrate a longer distance from the central predicting a lower detection rate in IB tests (Most et al., 2000; Kreitz et al., 2015). For example, in Newby and Rock’s (1998) study, the participants were invited to perform a “cross task” (judging which arm of the cross is longer) while the unexpected stimuli showed shortly in different positions. The participants were less likely to notice a further stimulus from the cross. Likewise, Most et al. (2000) conducted an IB study with a primary motion task guiding the participants to count the times that black letters “touch a baseline” on a screen while unexpected stimuli (crosses) are shown in different positions. The crosses positioned further from the “baseline” were more easily neglected by the participants.

However, different results are shown in a recent IB test undertaken by Kreitz et al. (2020) who explored the location effect of stimuli by grouping the stimuli’s positions into central and periphery areas, rather than testing their individual fixed positions. The participants were required to report the number of target stimuli (light grey triangles) placed in two clusters, whilst two unexpected stimuli (two squares) appeared around the clusters. One is between the clusters (the central area) and the other is outside the cluster (the periphery area). The researchers found significantly higher detection rates when the unexpected stimulus appeared in the periphery area rather than the central area. The researchers argued that this result may be caused by more visual attentional breadth being deployed in periphery areas than the central area. This study is consistent with a few other studies exploring the central inhibition effect that show the central stimuli being less detectable than those in the peripheral positions (Mack and Rock, 1998; Chen and Treisman, 2008; Thakral and Slotnick, 2010). The above studies indicate that stimuli’s location effect in IB tests should be considered from both perspectives of breadth and distances of attentional focus. In addition, some scholars claimed the non-existence of location effect in IB tests. The unexpected stimuli caused similar detection rates when they were in an individuals’ vision, regardless of their positions (Koivisto et al., 2004; Huang et al., 2012).

In conclusion, the research of IB test is to gain an understanding of the human being’s nature in attention division under different conditions. Researchers explored different stimuli with different research paradigms. The feature of stimuli (colour and size), the attention-demanding levels of primary tasks, and the location of the stimuli would affect the detection results in IB tests. The contradicting findings of the location effect in previous IB tests may be caused by the various primary tasks and the specific stimuli (occurrences) among these studies. Based on the existing research, though it is difficult to clarify the mechanism on how the location effect is associated with participants’ attention, the literature gives a clue that the location effect exists in many situations, which is the theoretical orientation of the present study.

### The Present Study

The purpose of this study is to explore the location effect of the off-seat behaviour in classrooms that is related to teachers’ attention to off-seat behaviour. It is an attempt to apply the approach of the IB test in the field of special education, which is rarely reported in the literature. Differing from previous studies that unexpected stimuli were presented when people were engaged in cognitively demanding tasks, the stimulus of the present study is the disappearance of an existing unit (student) in front of the participants who perform other classroom tasks. This experimental paradigm is innovative in IB research and will increase our knowledge about how “disappearing stimuli” are associated with people’s attention.

Grounded on previous studies, we hypothesized that a location effect exists in relation to the teachers’ identification of the unexpected off-seat behaviour when teachers are engaged in teaching tasks. Even though there is a controversial argument on the topic of the *central inhibition* or *central promotion* for detecting the unexpected stimuli, the evidence supporting the *central inhibition effect* seems to be more tenable. Enlightened by the study of Wood and Simons’s (2019) that participants showed attentional bias to above-oriented stimuli in the IB test, we speculated that there may also have some specifically oriented bias in the current study. Moreover, we hold the idea that the expectation would influence the teachers’ detection of off-seat behaviours (Beanland and Pammer, 2010). Therefore, we state the following hypotheses.

Hypothesis 1. A location effect in a classroom exists in relation to the teachers’ identification on off-seat behaviour.
Hypothesis 2. The *central inhibition effect* would be found in this IB test, that is, the detection rates of the off-seat behaviour that occurred in central seats would be lower than those in peripheral seats.
Hypothesis 3. The overt or covert cues of the off-seat behaviour’s occurrence would effectively improve the teachers’ detection of this behaviour.

## Materials and Methods

### Participants

For this study, 129 undergraduates who majored in education (pre-service teachers) were recruited from Zhejiang Normal University, China, which is a convenience sample. These participants were chosen with the following criteria: (a) completed internship experiences in special schools or preschools providing inclusive programs, and therefore these participants obtained basic knowledge and skills in special education and classroom management; (b) had no experiences of joining in similar IB experiments and were unaware of the fundamental purpose of the study; (c) have normal or corrected-to-normal vision. These participants were informed to retain the secret principle after the experiment, avoiding communication between one another.

Among the 129 participants, we excluded three participants’ data from the analysis because of their poor performance in the primary tasks of counting the number and labeling the positions of the students with their hands raised. These three participants did not pay full attention to the primary task (whose accuracy is less than 70%) that would impact the reliability of the results. In this regard, there were 126 participants in this study (M_age_ = 18.72 ± 0.723), and 92% were female.

### Materials

We used the E-Prime 3.0 software to write the IB task. To set up the experimental conditions appropriately, two elements were considered. Firstly, in Chinese special schools, the class group does not exceed 10 students and their seats are usually arranged in the pattern of three rows and three columns. Secondly, there are many situations where the teachers have difficulties allocating attention to all students in classrooms, and one typical context is that teachers need to address some students’ questions when students raise their hands. Therefore, the IB experiment task was designed to create a classroom scene on a computer screen in a similar pattern of three rows and three columns that includes nine students. The primary task for the participants to perform was to recognize the locations of the students putting their hands up.

**FIGURE 1:**
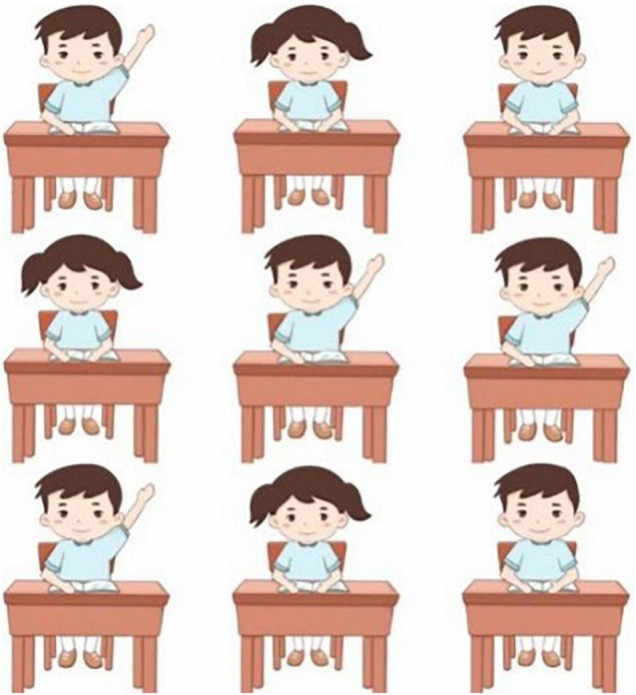
The scene of the classroom. Drawings created by Han Zhou. Reproduced with permission.

The classroom scene was illustrated by professional painters. The nine students sat on a chair behind a table as Chinese classroom management usually requires. As research suggests that conspicuous stimuli would attract more attention, the present study was then designed to make the appearance of “presenting students” be unified with the same hair and dressing styles and the same colour. This design was to exclude the effect from the feature of stimuli so as to focus on the investigation of the location effect in the classroom. The students who “appeared in the classroom” (including boys and girls) on the computer screen were presented in two styles: (1) sitting properly and listening to teachers; (2) raising hands to seek teachers’ attention. The unexpected occurrence of the study was the disappearance of one student who was positioned in different seats. Each combination of a student’s position was about 170 pixel (3.1°) × 234 pixel (4.3°). Figure 1 presents the scene of the classroom.

### Procedures

The ethics of this study were approved by the Ethics Committee of the Institute of Psychology, Zhejiang Normal University. This study was carried out in accordance with the Declaration of Helsinki (2013) and included informed consent, thus ensuring participants’ anonymity throughout the process and the option to quit at any moment. The tasks were presented on a laptop with a 14-inch monitor, a refresh rate of 60 Hz, and a screen resolution of 1,024 × 768 pixels. The participants were tested individually in the laboratory. They sat in front of a computer monitor with a viewing distance of approximately 60 cm. There were seven trials in the IB test, and each trial consisted of the following steps: (1) a tiny black central cue was displayed on the white screen for 1,000 ms; (2) nine students (including sitting properly and raising hands) in a classroom appeared at the center of the screen for 1,000 ms; (3) a visual mask with a black and grey pattern lasts for 1,300 ms; (4) operation interface to the primary task.

**FIGURE 2:**
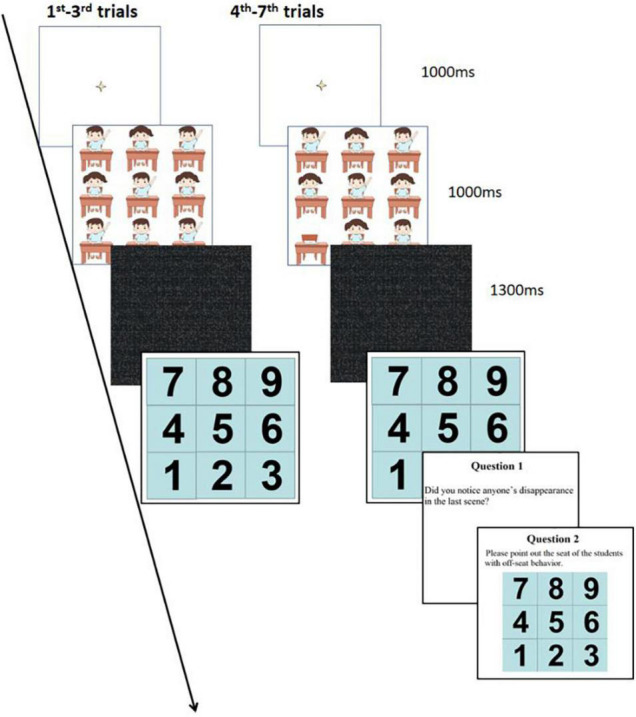
The flow diagram of the experiment. Drawings created by Han Zhou. Reproduced with permission.

The first three trials were formal trials in which the nine students, no matter sitting properly or raising hands, were presented in the scene of a classroom on the computer screen, and the participants only needed to complete the primary task, detecting who raised their hands and pointing out their locations. In each primary task, there were three to five targets (hand raising children) that randomly appeared on the screen. The fourth trial was a critical trial, where there were eight students presented and one student disappeared unexpectedly. The participants completed the primary task and then answered two extra questions to clarify whether they had detected the off-seat student. Question 1: Did you notice anyone disappearing in the last scene? Question 2: Please point out the seat of the student with the off-seat behaviour. If the participants were able to report that they noticed the missing student and select the right seat, then they were classified into the “NIB” (Non-Inattentional Blindness) group. The participants who failed to report the “missing student” or the location of the student were viewed as belonging to the IB group. The fifth trial was covert divided attentional trial because participants would have the anticipation of “missing student” and would pay attention to any follow-up stimuli though they were still asked to focus on the primary task. Participants who failed to notice and identify the position of the off-seat students in this trial were classified as DB_1_ (Divided Blindness 1) group, and on the contrary, they were sorted into the “NDB_1_” (Non-Divided Blindness 1) group. The sixth trial was overt divided attentional trial, in which the participants were instructed to pay attention to the “missing student” as well as complete the primary task. If the participants could not detect the position of the off-seat student, then they will be regarded as DB_2_ (Divided Blindness 2) group, or they will be classified as the “NDB_2_” (Non-Divided Blindness 2) group. The seventh trial was the focused attentional trial, where some prompts will be added to guide the participants to pay all their attention to the spot of the off-seat student. This trial was set to assess whether the participants were able to detect the disappeared student in the focused attention. Figure 2 shows the flow of the diagram. The positions of the seats were numbered (e.g., seat 1 and seat 2).

### Data Analysis

According to previous studies of IB (Klatt and Nerb, 2021; Yuan et al., 2021), we performed a statistical analysis to estimate the necessary sample size (G*Power 3.1.9.7, Düsseldorf, Germany). With a power of 0.80 and an alpha of 0.05, the projected sample size needed for medium effects of w = 0.40 was calculated, and it is atleast *N* = 94. The 126-sample size in the current study successfully met the requirement.

All the experimental data was coded and statistically analyzed using SPSS23.0. Chi-square tests combined with the binary logistic regression analyses that were performed to examine the location effect. Drawn on the theory of the *central inhibitory effect* of IB, we divided the seats into two categories: the center seats and the peripheral seats. The center seats included the seats of 2, 4, 5, 6, and 8 and the peripheral seats included the seats of 1, 3, 7, and 9.

## Results

### The Accuracy of the Primary Task

The mean accuracy of the primary task was 88.43 ± 3.75%. The t-test results revealed that there was no significant difference among the nine seats in terms of the accuracy of the primary task, *t*_(8)_ = –0.004, *p* > 0.05, which showed that the difficulty of the primary task in relation to the nine seats was manipulated reasonably in the study, thus avoiding irrelevant effects on the detection rates of unexpected stimulus.

### The Detection Rates of the Off-Seat Behaviour

**FIGURE 3:**
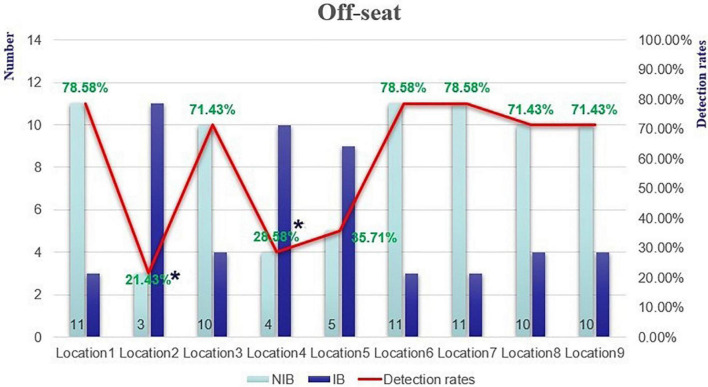
The number of detectors and detection rates of the off-seat behaviour. *means the *p* value is less than 0.05.

The number of detectors and the detection rates of the off-seat behaviour among the nine experimental seating locations in the critical trial are plotted in Figure 3, and the mean detection rates of off-seat behaviour were 59.5% in this trial. Meanwhile, the mean detection rates of the off-seat behaviour in the covert divided trial and overt divided trial were 88.81 and 94.44%, correspondingly.

A Chi-square test was conducted to examine the location effect on the detection rates of off-seat behaviour (Hypothesis 1). The results showed that there was a significant difference in the detection rates among nine seats, χ*^2^*(8, *N* = 126) = 26.089, *p* = 0.001, φ = 0.455. A binary logistic regression was analyzed to clarify which seats were more or less detectable for the off-seat behaviour, and the results showed that off-seat behaviour in seat 2 (*B* = –2.216, Wals = 6.340, *p* = 0.012, 95% CI [0.019, 0.612]) and seat 4 (*B* = –1.833, Wals = 4.798, *p* = 0.028, 95%CI [0.031, 0.825]) were significantly less noticeable than those in the other seats (see Figure 3). Please note that seat 2 was positioned below the central point of seat 5, and seat 4 was left to the central point.

Another Chi-square test was added to analyze the different location effects (central inhibitory effect) on the detection rates of off-seat behaviour (Hypothesis 2). The results showed that there was a significant difference in the detection rates between the center seats and the peripheral seats, χ*^2^*(1, *N* = 126) = 10.021, *p* = 0.002, φ = 0.282. Students with an off-seat behaviour were more likely to be detected when they were arranged in the peripheral seats than in the central positions.

A Cochran’s test was conducted to compare the detection rates in the two divided trials and a critical trial (Hypothesis 3). We found the detection rates in the two divided trials were significantly higher than in the critical trial, χ^2^(2, *N* = 126) = 58.857, *p* < 0.001, φ = 0.683.

## Discussion

This study adopted an IB task based on a semi-virtual class scene to explore the location effect in relation to the teachers’ detection of off-seat behaviour. The findings identified location effect in the classroom with “disappearing stimuli,” which extends the research paradigm of the IB test in educational settings. The results have implications for seat arrangements in classrooms for minimizing the potential risk of teachers’ IB on off-seat behaviour in special education.

The existence of the location effect in the classroom suggests that seats matter with regard to the teachers’ attention. This finding is in line with the research on spatial attention in the IB paradigm that unexpected occurrences that appeared in different spatial locations influence participants’ attentional distribution (Mack and Rock, 1998; Chen and Treisman, 2008; Thakral and Slotnick, 2010). Relevant research indicates that in addition to the factors of distance, the orientation of the stimuli affects the individual’s visual processing in IB tests as well (Wood and Simons’s, 2019). Specifically, the unexpected occurrences travelling horizontally above the fixation (the focus point that the participants need to pay attention to the primary task) were more likely to be noticed than those horizontally below (Wood and Simons’s, 2019). The result of seat 2 (below centre) showing less detection rate than the other seats seems to support this claim. However, the result of less attention on seat 4 (left to centre) contradicts the pseudoneglect phenomenon that individuals tend to exhibit a subtle attentional bias to the left space (Nicholls et al., 1999; Voyer et al., 2012). One possible reason is that the present IB study assigned participants with a primary task of high attentional load (three to five targets in each task). Therefore, there was extremely limited attention resources that remained for the unexpected stimulus to show the left-orientation bias. Relevant research also suggests that the left-orientation attentional bias is not stable when the attentional load increased, and the bias may shift to right-orientation because the interhemispheric rivalry results cause a more global decrease in right hemisphere activation that drives attention rightward (Peers et al., 2006; Dodds et al., 2008; Matthias et al., 2009; Newman et al., 2013). With limited studies, it is difficult to confirm a left or right bias of attention in IB tests, and further research is needed to confirm this issue.

Another significant finding of this study is that the detection rate of off-seat behaviour among peripheral seats is higher than that of the central seats. It resonates with the studies of a central inhibitory effect which shows a tendency of people’s attention shift, that is, a gradient increase of attentional suppression (allocation of attention) to target stimulus from the periphery to the center in visual perception. This tendency was found in both attentional condition, where participants’ attention was allocated to probe the target distractor (Bouma, 1973; Jonides, 1981; Juola et al., 1995; Goolkasian, 1999), and inattentional condition (such as IB paradigm) where distractor stimulus appeared suddenly (Mack and Rock, 1998). The theory of “inhibition of the return” might be the possible explanation for the detection advantages of the peripheral area. This theory suggests that the attentional focus is less likely to return to the previous attentional location after shifting away within a short duration [e.g., a few seconds; Klein (2000)]. Many participants experienced the focus shifting from central to marginal area in IB tests because participants are usually guided to focus on the fixation point in the central spot to make their attention prepared before formal trails (this study did this as well). This pre-experience facilitates the unexpected occurrence (stimulus) in the central area being ignored by the participants during their attention shifting away period. Another reason may relate to the design of the experiment that the peripheral locations of scratchable latex (3 rows * 3 columns) were far away from fixation (central point), which required participants to distribute broader attention resources to the process, so it took a slightly longer time for the participants to shift their attention back to the central area. This design increased the possibility for the unexpected occurrence (off-seat behaviour) in the peripheral locations being detected, while those in the center locations were suppressed.

Perhaps the most significant finding lies in the divided trials. The participants were more likely to detect an off-seat behaviour in the divided trial when holding the expectation on the unexpected stimuli. This result is consistent with previous studies that found higher detection rates in the divided trials as well. The participants would allocate more attentional resources to unexpected stimuli when having anticipants (Mack and Rock, 1998; Beanland and Pammer, 2010; Ward and Scholl, 2015; Zhang et al., 2022). This result suggests that teachers’ awareness and expectations about off-seat behaviour would assist them to identify this behaviour better in the classroom.

### Limitations and Implications

Although this study innovatively explored the location effect of IB in an educational context and achieved interesting findings, we should recognize its limitations. Firstly, the number of the total participants satisfies the statistic criterion, yet the participants who were assigned to identify the disappearing stimulus in different locations were limited. Secondly, the left-oriented (seat 4) and below-oriented inhibition (seat 2) identified in the study needs to be further clarified as there is no agreed theory in the field to endorse this finding. In addition to addressing these limits, future research needs to consider the IB tests to be undertaken in a real classroom. It would be meaningful to simulate a real classroom scenario using Virtual Reality technology to increase ecological validity (Huang et al., 2022; Seufert et al., 2022). Besides, the seat arrangements are not limited to the layout of scratchable latex (3 rows * 3 columns) as presented in this study. There are many other layouts which should be studied in the future.

Mindful of the above limitations, this study has important implications for classroom arrangement in the education of students with certain special needs. Based on our findings, some special-needs students may be more suitable to be placed in the peripheral locations, which are mainly distributed in the four corners of the classroom. The positive result of the divided trail advocates the necessity of enhancing pre-service and in-service teachers’ awareness of dangerous consequences of the off-seat behaviour, as this would increase teachers’ attention division for identifying this hazard more effectively.

## Data Availability Statement

The raw data supporting the conclusions of this article will be made available by the authors, without undue reservation.

## Ethics Statement

The studies involving human participants were reviewed and approved by Ethics Committee of the Institute of Psychology, Zhejiang Normal University. The patients/participants provided their written informed consent to participate in this study.

## Author Contributions

SC, XW, HZ, and JH designed the research and wrote the manuscript. SC and XW collected and analysed the data. All authors contributed to the article and approved the submitted version.

## Conflict of Interest

The authors declare that the research was conducted in the absence of any commercial or financial relationships that could be construed as a potential conflict of interest.

## Publisher’s Note

All claims expressed in this article are solely those of the authors and do not necessarily represent those of their affiliated organizations, or those of the publisher, the editors and the reviewers. Any product that may be evaluated in this article, or claim that may be made by its manufacturer, is not guaranteed or endorsed by the publisher.

## References

[B1] Allam F. C. Martin M. M. (2021). Issues and challenges in special education: a qualitative analysis from teacher’s perspective. *South. Asia Early Childh. J.* 10 37–49. 10.37134/saecj.vol10.1.4.2021

[B2] Alsius A. Navarra J. Campbell R. Soto-Faraco S. (2005). Audiovisual integration of speech falters under high attention demands. *Curr. Biol.* 15 839–843. 10.1016/j.cub.2005.03.046 15886102

[B3] Aspiranti K. B. Bebech A. Osiniak K. (2018). Incorporating a class-wide behavioral system to decrease disruptive behaviors in the inclusive classroom. *J. Catholic Educ.* 21 205–214. 10.15365/joce.2102102018

[B4] Beanland V. Pammer K. (2010). *Gorilla Watching: Effects of Exposure and Expectations on Inattentional Blindness. Gorilla Watching: Effects of Exposure and Expectations on Inattentional Blindness.* Sydney: Macquarie Centre for Cognitive Science, 12–20.

[B5] Bouma H. (1973). Visual interference in the parafoveal recognition of initial and final letters of words. *Vis. Res.* 13 762–782. 10.1016/0042-6989(73)90041-2 4706350

[B6] Castel A. D. Vendetti M. Holyoak K. J. (2012). Fire drill: inattentional blindness and amnesia for the location of fire extinguishers. *Atten. Percept. Psychophys.* 74 1391–1396. 10.3758/s13414-012-0355-3 22872548

[B7] Chen Y. (2013). *The effect of Size and Type on Inattentional Blindness.* Master’s thesis. Changchun: Northeast Normal University.

[B8] Chen Z. Treisman A. (2008). Distractor inhibition is more effective at a central than at a peripheral location. *Percept. Psychophys.* 70 1081–1091. 10.3758/PP.70.6.1081 18717393

[B9] Declaration of Helsinki (2013). Ethical principles for medical research involving human subjects. *JAMA* 310 2191–2194. 10.1001/jama.2013.281053 24141714

[B10] Dixon B. J. Daly M. J. Chan H. Vescan A. (2013). Surgeons blinded by enhanced navigation: the effect of augmented reality on attention. *Surg. Endosc.* 27 454–461. 10.1007/s00464-012-2457-3 22833264

[B11] Dodds C. van Belle J. Peers P. V. Dove A. Cusack R. Duncan J. (2008). The effects of time-on-task and concurrent cognitive load on normal visuospatial bias. *Neuropsychology* 22 545–552. 10.1037/0894-4105.22.4.545 18590365

[B12] Drew T. Võ M. L. H. Wolfe J. M. (2013). The invisible gorilla strikes again sustained inattentional blindness in expert observers. *Psychol. Sci.* 24 1848–1853. 10.1177/0956797613479386 23863753 PMC3964612

[B13] Feldon D. F. Callan G. Juth S. Jeong S. (2019). Cognitive load as motivational cost. *Educ. Psychol. Rev.* 31 319–337. 10.1007/s10648-019-09464-6

[B14] Finn J. D. (2019). Academic and non-cognitive effects of small classes. *Int. J. Educ. Res.* 96 125–135. 10.1016/j.ijer.2019.05.006

[B15] Furley P. Memmert D. Heller C. (2010). The dark side of visual awareness in sport: inattentional blindness in a real-world basketball task. *Attent. Percept. Psychophys.* 72 1327–1337. 10.3758/APP.72.5.1327 20601714

[B16] Goolkasian P. (1999). Retinal location and its effect on the spatial distribution of visual attention. *Am. J. Psychol.* 112 187–214. 10.2307/1423350 10696275

[B17] Ho M. H. Leung J. Mizubuti G. B. Contardi L. H. Chan M. Lo T. (2017). Inattentional blindness in anesthesiology: a simulation study. *J. Clin. Anesth.* 42 36–39. 10.1016/j.jclinane.2017.07.015 28802148

[B18] Huang S. S. Guo Y. N. Zheng X. F. (2012). The effects of unexpected stimulus’s meaning-fulness and position to Inattentional Blindness in college students. *Psychol. Dev. Educ.* 2 36–41. 10.16187/j.cnki.issn1001-4918.2012.02.012

[B19] Huang Y. Richter E. Kleickmann T. Richter D. (2022). Class size affects preservice teachers’ physiological and psychological stress reactions: an experiment in a virtual reality classroom. *Comput. Educ.* 184:104503. 10.1016/j.compedu.2022.104503

[B20] Huang Y. Richter E. Kleickmann T. Wiepke A. Richter D. (2021). Classroom complexity affects student teachers’ behavior in a VR classroom. *Comput. Educ.* 163:104100. 10.1016/j.compedu.2020.104100

[B21] Hughes-Hallett A. Mayer E. K. Marcus H. J. Pratt P. Mason S. Darzi W. (2015). Inattention blindness in surgery. *Surg. Endosc.* 29 3184–3189. 10.1007/s00464-014-4051-3 25582962

[B22] Jonides J. (1981). “Voluntary versus automatic control over the mind’s eye’s movement,” in *Attention and Performance IX*, eds Long J. B. Baddeley A. D. (Hillsdale, NJ: Erlbaum), 187–203.

[B23] Juola J. F. Koshino H. Warner C. B. (1995). Tradeoffs between attentional effects of spatial cues and abrupt onsets. *Percept. Psychophys.* 57 332–342. 10.3758/bf03213058 7770324

[B24] Kelly W. Jason C. Arien M. Tony R. (2018). Effects of canonical color, luminance, and orientation on sustained inattentional blindness for scenes. *Attent. Percept. Psychophys.* 80 1833–1846. 10.3758/s13414-018-1558-z 29987532

[B25] Klatt S. Nerb J. (2021). Position-specific attentional skills in team sports: a comparison between defensive and offensive football players. *Appl. Sci.* 11 5896. 10.3390/app11135896

[B26] Klein R. M. (2000). Inhibition of return. *Trends Cogn. Sci.* 4 138–147. 10740278 10.1016/s1364-6613(00)01452-2

[B27] Koivisto M. Hyönä J. Revonsuo A. (2004). The effects of eye movements, spatial attention, and stimulus features on inattentional blindness. *Vis. Res.* 44 3211–3221. 10.1016/j.visres.2004.07.026 15482807

[B28] Kreitz C. Furley P. Memmert D. Simons D. J. (2015). Inattentional blindness and individual differences in cognitive abilities. *PLoS One* 10:e0134675. 10.1371/journal.pone.0134675 26258545 PMC4530948

[B29] Kreitz C. Hüttermann S. Memmert D. (2020). Distance is relative: inattentional blindness critically depends on the breadth of the attentional focus. *Conscious. Cogn.* 78:102878. 10.1016/j.concog.2020.102878 31978756

[B30] Lever C. (2014). *Understanding Challenging Behavior in Inclusive Classrooms.* Harlow: Pearson Longman.

[B31] Mack A. Rock I. (1998). *Inattentional Blindness.* Cambridge: MIT Press.

[B32] Matthias E. Bublak P. Costa A. Müller H. J. Schneider W. X. Finke K. (2009). Attentional and sensory effects of lowered levels of intrinsic alertness. *Neuropsychologia* 47 3255–3264. 10.1016/j.neuropsychologia.2009.08.004 19682470

[B33] Most S. B. Simons D. J. Scholl B. J. Chabris C. F. (2000). Sustained inattentional blindness: the role of location in the detection of unexpected dynamic events. *Psyche* 6:13.

[B34] Newby E. A. Rock I. (1998). Inattentional blindness as a function of proximity to the focus of attention. *Perception* 27 1025–1040. 10.1068/p271025 10341933

[B35] Newman D. P. O’Connell R. G. Bellgrove M. A. (2013). Linking time-on-task, spatial bias and hemispheric activation asymmetry: a neural correlate of rightward attention drift. *Neuropsychologia* 51 1212–1223. 10.1016/j.neuropsychologia.2013.03.027 23583973

[B36] Nicholls M. E. R. Bradshaw J. L. Mattingley J. B. (1999). Free-viewing perceptual asymmetries for the judgement of brightness, numerosity and size. *Neuropsy-chologia* 37 307–314. 10.1016/S0028-3932(98)00074-8 10199644

[B37] Pammer K. Blink C. (2013). Attentional differences in driving judgments for country and city scenes: semantic congruency in inattentional blindness. *Accid. Anal. Prevent.* 50 955–963. 10.1016/j.aap.2012.07.026 22975367

[B38] Patterson R. T. Steven T. (2009). The effects of teacher-student small talk on out-of-seat behavior. *Educ. Treat. Child.* 32 167–174. 10.1353/etc.0.0048 34409987

[B39] Peers P. V. Cusack R. Duncan J. (2006). Modulation of spatial bias in the dual task paradigm: evidence from patients with unilateral parietal lesions and controls. *Neuropsychologia* 44 1325–1335. 10.1016/j.neuropsychologia.2006.01.033 16522325

[B40] Phillips K. Downer J. (2017). Classroom context and years of teaching experience as predictors of misalignment on ratings of preschoolers’ classroom engagement. *Early Educ. Dev.* 28 343–367. 10.1080/10409289.2016.1218730

[B41] Polirstok S. (2015). Classroom management strategies for inclusive classrooms. *Creat. Educ.* 6 927–933. 10.4236/ce.2015.610094

[B42] Prieto L. P. Sharma K. Kidzinski Ł Dillenbourg P. (2018). Orchestration load indicators and patterns: in-the-wild studies using mobile eye-tracking. *IEEE Trans. Learn. Technol.* 11 216–229. 10.1109/TLT.2017.2690687

[B43] Qin J. K. Chen K. Q. (2011). The influence of the unexpected stimulus’ color and shape on Inattentional Blindness. *Psychol. Res.* 4 38–43.

[B44] Seufert C. Oberdörfer S. Roth A. Grafe S. Lugrin J. L. Latoschik M. E. (2022). Classroom management competency enhancement for student teachers using a fully immersive virtual classroom. *Comput. Educ.* 179:104410. 10.1016/j.compedu.2021.104410

[B45] Simons D. J. Chabris C. F. (1999). Gorillas in our midst: sustained inattentional blindness for dynamic events. *Perception* 28 1059–1074. 10.1068/p2952 10694957

[B46] Thakral P. P. Slotnick S. D. (2010). Attentional inhibition mediates inattentional blindness. *Conscious. Cogn.* 19 636–643. 10.1016/j.concog.2010.02.002 20227894

[B47] Voyer D. Voyer S. D. Tramonte L. (2012). Free-viewing laterality tasks: a multilevel meta-analysis. *Neuropsychology* 26 551–567. 10.1037/a0028631 22731609

[B48] Wang F. X. Lu Y. L. Duan Z. H. Zhou Z. K. (2013). Teachers’ perception of students’ classroom behaviors: an eye movements study. *Psychol. Dev. Educ.* 4 391–399. 10.16187/j.cnki.issn1001-4918.2013.04.004

[B49] Ward E. J. Scholl B. J. (2015). Inattentional blindness reflects limitations on perception, not memory: evidence from repeated failures of awareness. *Psychon. Bull. Rev.* 22 722–727. 10.1167/15.12.182 25515671

[B50] Wiseman R. Watt C. (2015). And now for something completely different: inattentional blindness during a monty python’s flying circus sketch. *i-Perception* 6 38–40. 10.1068/i0706sas 26034570 PMC4441020

[B51] Wolff C. E. Jarodzka H. Niek V. D. B. Boshuizen H. P. A. (2016). Teacher vision: expert and novice teachers’ perception of problematic classroom management scenes. *Instruct. Sci.* 44, 243–265. 10.1007/s11251-016-9367-z

[B52] Wood K. Simons D. J. (2019). The spatial allocation of attention in an interactive environment. *Cogn. Res. Princ. Imlic.* 4:13. 10.1186/s41235-019-0164-5 PMC6470233 30997621

[B53] Yang J. Zhu Z. S. Cao S. Q. (2012). A functional behavior assessment-based case study of the seat-leaving behavior in the early childhood classroom. *Chin. J. Spec. Educ.* 11 18–23.

[B54] Yuan L. Y. Chang R. S. Ma J. F. (2021). Regular schematic start training in the process of drivers’ selective attention. *Acta Psychol. Sin.* 12 1310–1320.

[B55] Zhang H. Wang J. L. Liu Y. Yan C. C. Ye X. H. (2022). Threat-relevant stimuli cannot be better detected by preschoolers in an inattentional blindness task. *Psychol. Res.* 86 823–830. 10.1007/s00426-021-01530-5 34018023

